# Intrasphenoid septations inserted into the internal carotid arteries: a frequent and risky relationship in transsphenoidal surgeries^☆^

**DOI:** 10.1016/j.bjorl.2016.02.007

**Published:** 2016-04-22

**Authors:** Clauder Oliveira Ramalho, Horacio Armando Marenco, Francisco de Assis Vaz Guimarães Filho, Marcos Devanir Silva da Costa, Bruno Fernandes de Oliveira Santos, Rodrigo de Paula Santos, Samuel Tau Zymberg

**Affiliations:** a Universidade Federal de São Paulo (UNIFESP), Departamento de Neurocirurgia, São Paulo, SP, Brazil; b Universidade Federal de São Paulo (UNIFESP), Programa de Pós-graduação do Departamento de Otorrinolaringologia e Cirurgia de Cabeça e Pescoço, São Paulo, SP, Brazil; c Universidade Federal de São Paulo (UNIFESP), São Paulo, SP, Brazil; d Universidade Federal de São Paulo (UNIFESP), Departamento de Otorrinolaringologia, São Paulo, SP, Brazil

**Keywords:** Sphenoid sinus, Sphenoid septations, Skull base, Transsphenoidal surgery, Expanded endonasal approach, Seio esfenoidal, Septações esfenoidais, Base do crânio, Cirurgia transesfenoidal, Abordagem endonasal ampliada

## Abstract

**Introduction:**

When an expanded endonasal transsphenoidal surgical approach is performed, intrasphenoid septations must be completely resected. If these structures are close to the internal carotid artery (ICA), then their manipulation might cause vascular injury.

**Objective:**

The objective of this study is to describe the frequency of intrasphenoid septations in the internal carotid artery protuberance (ICAp).

**Methods:**

Computed tomography (CT) scans of 421 patients were analysed. Intrasphenoid septations (classified as intersphenoid or accessory) and their relationship to the ICAp were described. Additionally, a sphenoid sinus classification was performed based on their degree of pneumatisation to determine whether a difference exists in the frequency of intrasphenoid septations inserted into ICAp with regard to sinus type.

**Results:**

The patient mean age was 39 ± 21.4 years. Overall, 219 patients (52%) had septations in the ICAp; 359 patients (85.3%) had intersphenoid septations; of the latter, 135 (37.6%) had septations in the ICAp. This frequency was higher among patients with sphenoid sinus type 4 or 5 (44.7% and 43.5%, respectively). Accessory septations were found in 255 patients (60.6%); 140 of these septations (54.9%) were in the ICAp. Among 351 patients with types 3, 4 or 5 sphenoid sinuses (i.e., only well-pneumatised sphenoid sinuses), 219 (62.4%) had septations in the ICAp. These frequencies are higher than those reported in most previous studies.

**Conclusion:**

The frequency of intrasphenoid septations in the ICAp found is considerable. It is higher among patients with more pneumatised sinuses. This finding justifies an appropriate pre-operative study, and careful attention must be paid during transsphenoidal surgery.

## Resumo

**Introdução:**

Quando uma abordagem cirúrgica transesfenoidal endonasal ampliada é realizada, septações intraesfenoidais devem ser completamente ressecadas. Se estas estruturas estiverem próximas à artéria carótida interna (ACI), a manipulação pode causar lesão vascular.

**Objetivo:**

O objetivo deste estudo foi descrever a frequência de septações intraesfenoidais na protuberância da artéria carótida interna (pACI).

**Método:**

Exames de tomografia computadorizada (TC) de 421 pacientes foram analisados. As septações intraesfenoidais (classificadas como interesfenoidais ou acessórias) e sua relação com a pACI foram descritas. Além disso, uma classificação do seio esfenoidal foi realizada com base no seu grau de pneumatização para determinar se existe uma diferença na frequência de septações intraesfenoidais inseridas em pACI em relação ao tipo de seio.

**Resultados:**

Pacientes com idade média de 39 ± 21,4 anos foram incluídos. No geral, 219 pacientes (52%) apresentavam septações na pACI; 359 (85,3%) tinham septações interesfenoidais; 135 (37,6%) com septações na pACI. Esta frequência foi maior entre os pacientes com seio esfenoidal tipo 4 ou 5 (44,7 e 43,5%, respectivamente). As septações acessórias foram encontradas em 255 doentes (60,6%); 140 dessas septações (54,9%) estavam na pACI. Entre 351 pacientes com seios esfenoidais tipos 3, 4 ou 5 (isto é, apenas seios esfenoidais bem-pneumatizados), 219 (62,4%) tinham septações na pACI. Estas frequências são superiores às relatadas na maioria dos estudos.

**Conclusão:**

A frequência de septações intraesfenoidais na pACI encontrada é considerável, sendo maior entre pacientes com seios mais pneumatizados. Este achado justifica um estudo pré-operatório adequado e uma atenção especial deve ser dada durante a cirurgia transesfenoidal.

## Introduction

Transnasal transsphenoidal surgery has developed significantly over recent decades. The cooperative work between neurosurgeons and ear, neck and throat surgeons has been essential for this development. The introduction of the endoscope was another landmark. Compared with the microscope, the endoscope enabled additional expansion of this surgical technique, thereby increasing the possibility of resecting lesions not otherwise eligible for transnasal transsphenoidal surgery.^1^ With the emergence of the endoscopic expanded endonasal approach, areas such as the clivus, the petrous bone, the middle cranial fossa and the infratemporal fossa became accessible.^2^ An extensive sphenoidotomy with septation resection is necessary to create an adequate surgical corridor.^3^

**Figure 1:**
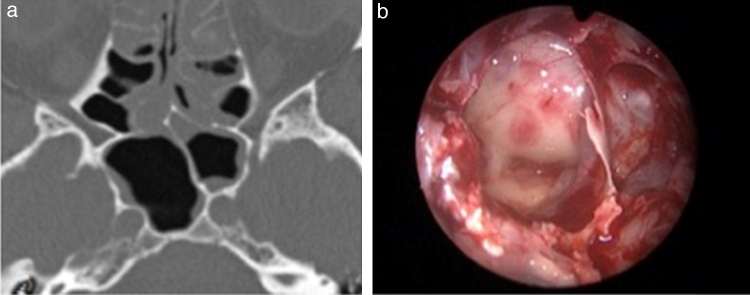
(a) CT scan axial view of an intersphenoid septation in the left internal carotid artery protuberance (ICAp) and (b) intraoperative view (0° endoscope) showing the same septation in the left ICAp.

Intrasphenoid septations are bony structures found in the sphenoid sinus with several anatomical conformations. Because they are located in the sinus walls, they are often adjacent to surrounding structures, especially the internal carotid artery (ICA), which can increase the risk of expanded transsphenoidal surgeries during septation resection (Fig. 1).

ICA injury is one of the most dramatic intraoperative complications. This injury can lead to a challenging surgical scenario featuring rapid blood loss that can result in patient exsanguination.^4^ An appropriate pre-operative radiologic evaluation of the sphenoid sinus and its septations is necessary to prevent this complication.

Previous articles have described the frequency of intrasphenoid septations in the ICA protuberance (ICAp).^1^, ^2^, ^3^, ^4^, ^5^ The majority of these articles have found fewer intrasphenoid septations than our surgical and pre-operative findings. The current article describes the frequency of intrasphenoid septations in the ICAp among a sample of 421 patients analysed using computed tomography (CT) scans and compares these findings with those of previous studies.

## Methods

### Sample and selection criteria

We searched the database of the department of radiology of a hospital institution from January 2010 to April 2013 for patients who underwent CT scans of the skull base. Individuals with a previous history of paranasal sinus disease or endonasal surgery were excluded. A total of 421 patients were selected. Informed consent was obtained from all individual participants included in the study.

All patients underwent a CT scan with skull base sections using the Brilliance CT 64 system (Philips, 2004). The scan was performed with 20 × 0.625 collimation, a pitch of 0.348, a matrix of 512; 200 mm of field of view. Section thickness ranged from 0.6 to 1 mm. The obtained data were transferred to the Extended Brilliance Workspace (Philips Medical System), where the images were reconstructed in the axial, coronal, and sagittal planes.

### Septation type definition and its relationship with ICAp

**Figure 2:**
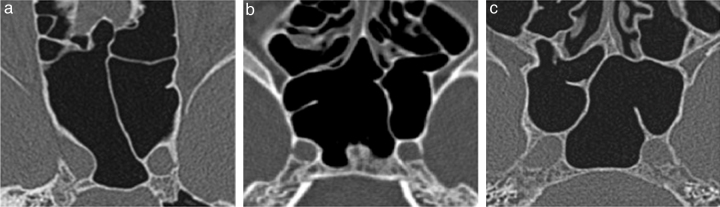
Septation type definition and its relationship with the internal carotid artery protuberance (ICAp). Axial reformatted images obtained from CT data scanning. (a) Intersphenoid septation in the left ICAp. (b) Intersphenoid septation in the left ICAp and an accessory septation in the left sinus wall. (c) Intersphenoid septation in the right ICAp and an accessory septation in the left ICAp.

Intrasphenoid septations were classified as intersphenoid when they (1) were longitudinal and in a median or paramedian location and (2) separated the cavity into two non-communicating compartments from the anterior to the posterior sinus wall. A septation was defined as an accessory when it did not follow all of the intersphenoid sinus patterns (Fig. 2).

To consider the true relationship between septation and ICAp, a CT section had to clearly show a septation in this structure (Fig. 2).

### Sinus classification

**Figure 3:**
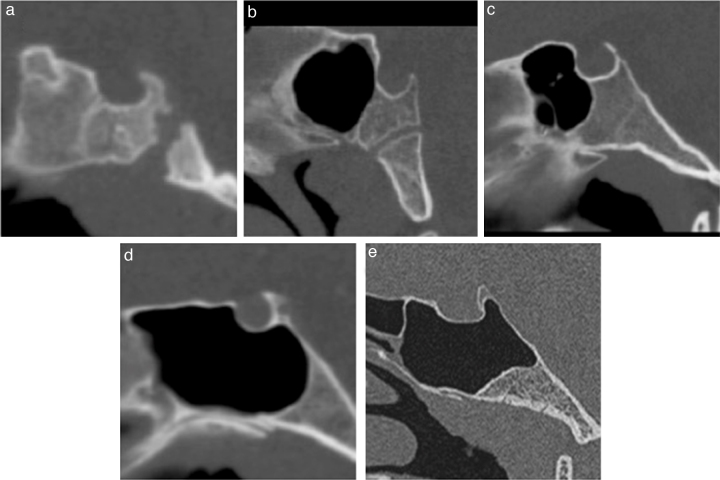
Sinus classification. Midsagittal reformatted images obtained from CT scans. (a–e) Types 1 through 5 sphenoid sinuses, respectively.

Sphenoid sinuses were classified based on their degree of pneumatisation, which was established by the spatial relationship of the sinus posterior wall and the anterior and posterior walls of the sella turcica. Sinuses were classified as follows: those with an absence of aeration or minimal aeration were classified as type 1; those with their posterior wall in a position rostral to the sella anterior wall were classified as type 2; those with their posterior wall between the sella anterior and posterior walls were classified as type 3; those with their posterior wall reaching the posterior wall of the sella were classified as type 4; and those with posterior clinoid aeration were classified as type 5. The purpose of this analysis was to assess whether the frequency of septation in the ICAp differed across patients with disparate sinus types (Fig. 3).

### Statistical analyses

Categorical variables were described by numbers of cases and percentages. Groups were compared using the *z*-test for proportions and either the chi-square test or Fisher's exact test, as appropriate. Continuous variables were characterised as either the mean ± standard deviation or the median and interquartile range depending on normality; between-group comparisons were made using Student's *t*-test or the Kruskal–Wallis test, respectively. A result was considered significant when *p* < 0.05. Statistical analyses were conducted with the SPSS 17 (Chicago, IL, USA).

### Research protocol

The ethics committee approved the research protocol (document number 186.717).

## Results

We identified 189 male and 232 female patients (mean age: 39 ± 21.4 years). The most frequent type of sinus was type 4 (61% of cases) (Table 1).

**Table 1:** Intersphenoid and accessory septations by sinus type.

	Sinus type				
	1	2	3	4	5
Total (%)	26 (6.2)	44 (10.5)	71 (16.9)	257 (61)	23 (5.5)
Mean age (interquartile range)	4 (8)^a^	35 (51)	43 (34)	42 (30)	46 (28)
Males	14 (53.8)	24 (54.5)	33 (46.5)	104 (40.5)	14 (60.9)
Intersphenoid septation	–	37 (84.1)	66 (93)	237 (92.2)	19 (82.6)
Intersphenoid septation in the internal carotid artery protuberance (ICAp)	–	–	10 (14.1)	115 (44.7)	10 (43.5)
Accessory septation	–	11 (25)	45 (63.4)	181 (70.4)	18 (78.3)
Accessory septation in the ICAp	–	–	18 (25.4)	110 (42.8)	12 (52.2)

a Median significantly differs from the others (*p* < 0.05).

A total of 359 patients (85.3%) had intersphenoid septations. Out of these septations, 135 (37.6%) were found in the ICAp. Totals of 44.7% and 43.5% of patients with type 4 and 5 sinuses had intersphenoid septations in the ICAp, respectively; only 14.1% of patients with type 3 sinuses had these septations in the ICAp. Patients with types 1 or 2 sinuses did not have ICAp-adjacent septations.

Accessory septations were found in 255 patients (60.6%). These septations were present in only one-quarter of patients with type 2 sinuses, whereas most of those with types 3, 4 or 5 sinuses had septations. Septations were located in the ICAp of 140 patients (54.9% of those with accessory septations; 25.4%, 42.8% and 52.2% for types 3, 4 and 5, respectively). The maximum numbers found were 2, 3, 5, and 4 for types 2, 3, 4, and 5, respectively, whereas the maximum numbers found in the ICAp were 2, 3, and 3 for types 3, 4, and 5, respectively.

The number of patients with septation in the ICAp, regardless of type, was 219 (52%). Those patients were older than those without septations (43 ± 18 vs. 34 ± 23; *p* < 0.0001); no significant difference was observed with regard to sex (females comprised 55% of all cases in both groups, *p* = 0.963). Patients with types 4 and 5 sphenoid sinuses were more common than those with type 3 (Table 2).

**Table 2:** Sphenoid septation in the internal carotid artery protuberance by sinus type.

	Total	%	
Type 1	–	–	
Type 2	–	–	
Type 3	23	32.4%	a
Type 4	179	69.6%	b
Type 5	17	73.9%	b

Proportions identified with different letters are significantly different according to the *z*-test for proportions (*p* < 0.05).

Of the 351 patients with type 3, 4 or 5 sphenoid sinuses, 219 (62.4%) had septations in the ICAp. A total of 322 patients (91.7%) had intersphenoid septations; of these patients, 135 (41.9%) had septations in the ICAp. Of the 244 patients with at least one accessory septation, 140 (57.4%) had a septation in the ICAp.

## Discussion

The expanded endonasal transsphenoidal approach marked a breakthrough in skull base surgery. With its development, lesions previously inaccessible using the conventional endonasal route (e.g., those in the cavernous sinus, the planum sphenoidale, the middle cranial fossa, Meckel's cave, the suprasellar region and the clivus) could be accessed.^2^ To obtain appropriate exposure and accommodate the surgical endoscopic instruments, a wide sphenoid sinus opening is required with intrasphenoid septation resection.^3^

Intrasphenoid septations are naturally occurring bony structures inside the sphenoid sinus that divide it into compartments. They are divided into intersphenoid and accessory septations. The association with fusion lines between ossification centres (synchondrosis) and the septation positions might explain their origin.^6^, ^7^ In general, one or more intersphenoid septations are present. They show great variability; therefore, they typically create two asymmetric compartments: right and left. Accessory septations occur in different positions and are also common. Both can be found in structures adjacent to the sphenoid sinus increasing the risk of neurovascular damage during surgery, especially when they are located in the ICAp. Cope previously described this complication in 1917.^6^

In our study, CT scans revealed that 219 patients (52%) had septations in the ICAp. Among patients with type 3, 4, or 5 sinuses (i.e., well-pneumatised sphenoid sinuses), this prevalence was even higher (62.4%).

Our data contrast with those of previous papers showing a smaller prevalence.^5^, ^8^, ^9^, ^10^, ^11^, ^12^ However, Fernandez-Miranda et al. showed radiologic prevalence of 85% among patients with at least one septation in the ICAp.^3^

Renn and Rhoton found intersphenoid septations next to the ICA channel in 32% of cadavers.^5^ Sethi described intersphenoid septations in the ICAp in 40% of 30 cadavers in an endoscopic study in 1995.^8^ Unal et al. and Abdullah et al. reported 30% and 31% of septations of the sphenoid sinus attached to the wall of the ICA, respectively, using CT scans.^10^, ^12^

Elwany et al. found that 12.9% of patients had septations in the bone surrounding the ICAp in an endoscopic study with 93 cadavers.^9^ Hamid et al. showed frequencies of 4.7% and 6.75% for intersphenoid and accessory septations in the ICAp, respectively.^11^ Both of these studies represent the lowest frequencies in the literature.

The current findings support the need for a pre-operative radiologic study on intrasphenoid septations. Appropriate knowledge concerning their position and their relationship to surrounding structures might significantly decrease the risk of surgery catastrophes due to vascular injuries.

Pre-operative CT scans are the radiological evaluation of choice because they adequately visualise bone structures. The analysis of the axial, coronal, and sagittal planes as well as their 3 dimensional reconstruction ability allow radiologists to accurately determine whether a septation is close to the structures surrounding the sphenoid sinus (e.g., the ICA).^3^, ^13^, ^14^, ^15^, ^16^

With regard to sinus classification, patients with type 4 or 5 sinuses were more likely to have septations in the ICAp than patients with type 3 sinuses. The aeration process that the sphenoid body undergoes explains this result. When the ICA is pronounced, it can substantially bulge into the pneumatised sinus, thereby increasing the area that is susceptible to a septation attachment.^13^

The fact that sinuses are more pneumatised in older people might explain the higher mean age of the patients who showed a relationship between septations and the ICAp.^17^

The present study describes the anatomical findings of a large, multiracial population. To our knowledge, it is the largest series regarding this issue.

## Conclusion

The high frequency of intrasphenoid septations in the ICAp requires an appropriate pre-operative radiologic study. Furthermore, careful attention should be paid during transsphenoidal surgery to reduce potentially serious vascular injuries.

## Conflicts of interest

The authors declare no conflicts of interest.
